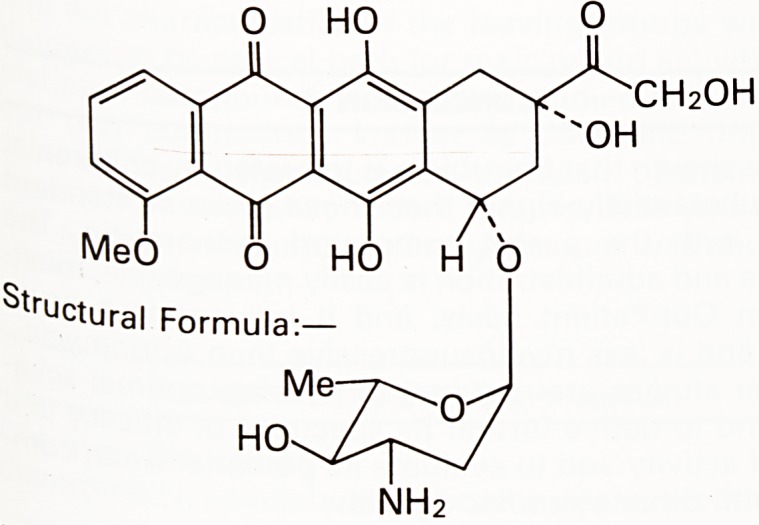# Epirubicin

**Published:** 1988

**Authors:** J. Cornish, A. Oakhill, M. G. Mott

**Affiliations:** Bristol Children's Hospital; Bristol Children's Hospital; Bristol Children's Hospital


### Epirubicin in Childhood Cancer

**J. Cornish, A. Oakhill, M. G. Mott.**
Bristol Children's Hospital

Analogue development is an intensively pursued goal. The rationale behind this is the selection of agents with an improved therapeutic index with respect to the parent compound but with an enhanced spectrum of activity, and reduced toxicity.

Epirubicin is a new anthracycline antibiotic, synthesised in an effort to find a cytotoxic agent with a better therapeutic index than that of Adriamycin – the parent drug with which all clinical oncologists are familiar.

Epirubicin differs from Adriamycin in the configuration of the hydroxyl group in the C4 position on the amino sugar moiety. This simple structural alteration of the molecule has resulted in an anticancer agent with:—

1. Modified pharmacokinetics, particularly with regard to its metabolism and routes of elimination.

2. The same activity as Adriamycin in Adriamycin-responsive tumours.

3. Activity against some Adriamycin-resistant tumours, such as pancreatic carcinoma, suggesting that it may have a broader spectrum of activity than the parent compound.

4. A lower incidence and severity of undesirable side effects, particularly cardiotoxicity.

Epirubicin has been used at the Bristol Children's Hospital since February, 1986, either as a single agent or in combination with other cytotoxics. It is appropriate, however, before describing our experience, to consider first the properties of the drug itself.

The chemical name is:— (75:95) -9-hydroxyacetyl-4-methoxy-7,8,9,10, tetrahydro-6,7,9,11-tetrahydroxy-7-0-(2,3,6-trideoxy-3-amino-x-L-arabino-Hexopyranosyl)-5, 12-naphthacenedione, hydrochloride.

Structural Formula:—

It is a red-orange crystalline powder, and is prepared for intravenous administration by reconstitution with water for injection or Sodium Chloride. The reconstituted solution is stable for 24 hours at room temperature or 48 hours if kept in a refrigerator, and should be protected from direct light. We administer Epirubicin in a free-running infusion of 5% Dextrose/Saline over up to one hour. This allows for the flushing through of the vein, and minimises the risk of extravasation with consequent severe necrosis to surrounding tissues.

**Mechanisms of Action**

The cytotoxic effects of Epirubicin are similar to adriamycin, with intercalation into the DNA double helix structure. This results in damage to DNA and interference with the synthesis of DNA, RNA and proteins. Such irreversible damage to the DNA plays a major contributory role in the events that result in cell death.

Epirubicin has increased lipid solubility compared with Adriamycin, which results in a higher influx rate and cellular accumulation of this analogue. It also undergoes more extensive metabolism to inactive or more rapidly excreted metabolites.

Tumour cells exhibit a lower Ph value (because of their higher lactate content) thus allowing for more dissociated drug to act upon the cellular mechanisms. This may account for the reduced toxicity of Epirubicin without loss of cytotoxicity.

Epirubicin also interferes with the integrity and activity of cell-membranes, and maximal cell kill occurs during the S phase of the cell cycle.

**Pharmacokinetics and Metabolism**

The pharmacokinetics of Epirubicin have been studied in cancer patients after rapid intravenous administration, and the highest uptake appears to be in the tumour, surrounding areas, and gall bladder, the lowest being in adipose tissues, muscles, spleen and serous membranes. The distribution does not differ substantially from that of Adriamycin, although, as suggested by animal data, the tissue concentrations of Epirubicin are lower, and it is less retained in the heart.

Following intravenous administration, there is a rapid distribution phase and prolonged elimination phase consistent with extensive drug retention within the peripheral tissues and gradual release thereafter.

Epirubicin, like Adriamycin, is primarily eliminated by the hepatobiliary system (40% of the administered dose in 4 days). The terminal half-life of Epirubicin is 30–40 hours in contrast with a 40–70 hour elimination half-life for Adriamycin; it therefore has a shorter terminal half-life and higher plasma clearance.

Epirubicin is characterised not only by a faster elimination than Adriamycin, but also an additional metabolic pathway. Whereas Adriamycin has only one metabolite, several metabolites can be detected in plasma and urine after Epirubicin administration. In particular, a unique formation of glucuronides have been detected which have not been demonstrated for Adriamycin and may account for the faster elimination of Epirubicin.

**Phase I and Phase II Studies**

Phase I Studies are designed to define the toxicological pattern of a drug and to determine the maximum tolerated dose in men.

Phase I Studies carried out in two major centres, the National Tumour Institute in Milan, and the Memorial Sloan-Kettering Cancer Centre, New York, have shown a reduced incidence of acute toxicities such as vomiting, mucositis and neutropenia for Epirubicin. In the U.S., studies demonstrated a remarkable range for dose-limiting myelosuppression of Epirubicin, with doses escalating to 135 mgs/m2, without major myelotoxocity (our study raised this level even higher). A similar earlier study carried out by the same investigators in the same institution had demonstrated that only a few patients receiving Adriamycin could tolerate doses of 90 mg/m2.

| *Diagnosis* | *No of Patients* | *Relapse* | *Previous Chemo* |
|---|---|---|---|
| Ewings | 4 | 1 | 1 |
| Neuroblastoma | 3 | 1 | 1 |
| Osteosarcoma | 3* | — | — |
| Rhabdomyosarcoma | 2 | — | — |
| Wilms | 1 | 1 | 1 |
| Testicular Teratoma | 1 | 1 | 1 |

*Chemotherapy schedule changed because no evidence of chemotherapy effect at tumour excision.

Similarly, statistical analysis indicated that there was a linear dose-dependant relationship for acute cardiotoxicity of Epirubicin, as with Adriamycin, but Epirubicin has a lower toxic effect on myocardial contractility than Adriamycin.

The Phase II Studies were disease-orientated and numerous, and designed principally to evaluate the spectrum of anticancer activity while further investigating toxicity. Broadly speaking, it was found that Epirubicin had equivalent efficacy to its parent compound, Adriamycin with reduced toxicity.

Our own study using Epirubicin started in February 1986. The general principles of cancer chemotherapy apply in the treatment of paediatric malignancy, although the effectiveness of these agents depends even more on achieving the maximum tolerated dosage without prohibitive toxicity. Most children are able to tolerate higher doses of chemotherapeutic agents than adults.

From February 1986 to January 1987, 14 patients received 37 courses of Epirubicin, 33 of them at a dose of 150 mg/m2. The dose was modified in 4 courses, the reasons being:—

1. Fever and prolonged neutropenia.
2. Previous radiation (2 courses).
3. Medical reluctance (first dose given at the Children's Hospital in February 1986, with previous published data suggesting a maximum of 90 mgs/m2 as a single dose).

The Epirubicin was administered intravenously in N Saline over one hour, usually following a single IV dose of Vincristine, and was part of a regime, the Bristol Children's Hospital Resistant Tumour Protocol.

In this protocol, the Epirubicin and Vincristine are given as part of a 9-week cycle that includes Ifosfamide and VP16, and Carboplatinum, alternating at 3 weekly intervals, usually for 5 complete cycles or a total of 1 year. The patients characteristics are tabulated above and cover the typical range of paediatric resistant tumours.

We found that the degree of myelosuppression with Epirubicin was acceptable. The nadir counts for neutrophils and platelets occurred between the 8th and 15th day after administration, usually at day 10, and neutropenia was observed more often than thrombocytopenia. There was, however, complete bone marrow recovery by the third week after administration, and there were no delays in giving the next drug in the cycle.

| Nadir values | *Range* | *Mean* | *Median* |
|---|---|---|---|
| Total WBC | 0.6–11.7 | 2.33 | 1.7 |
| Neutrophils | 0–4.8 | 1.13 | 0.75 |
| Platelets | 44–445 | 168 | 155 |

Neutropenia of $<1\times10^9$/L occurred in 22 of the 37 courses, although there was only one admission to hospital for treatment of a neutropenic fever.

7 of the 37 courses resulted in a thrombocytopenia of $<100\times10^9$/L, but no patient needed support with platelet transfusions, and there were no bleeding manifestations.

There was a low incidence of anaemia, and we did not consider this a significant problem.

Gastrointestinal effects were frequently reported, the most common being nausea and vomiting. All our patients were treated, and some pre-treated, with antiemetics, and vomiting on this regime was nil to moderate. Alopecia was universal, but other drugs were also contributory to this.

Thrombophlebitis was seen in all patients who did not have central venous catheters.

Cardiotoxicity was noted in only one patient, a young man undergoing re-treatment for a relapse of his Ewings tumour, who had previously been treated with Adriamycin. He developed an abnormal echocardiogram at 300 mgs/m2 of Epirubicin,and we stopped at this dose. All patients had an echo performed before each dose of Epirubicin, and so far we have reached a total cumulative dose of 750 mgs/m2 in one young patient, with no signs of cardiotoxicity.

### CONCLUSION

We have shown that Epirubicin is tolerated by children at doses substantially higher than those given as standard therapy with the parent compound, Adriamycin. The schedule and administration is easily manageable, mostly on an Out-Patient basis, and it induces less acute toxicity and is less myelosuppressive than Adriamycin.

Further studies are required to develop optimal schedules, and to define further its spectrum of efficacy and range of activity and to evaluate its potential when combined with other cytotoxic agents.

## Figures and Tables

**Figure f1:**